# Cell-Cycle-Related and Expression Elevated Protein in Tumor Upregulates the Antioxidant Genes via Activation of NF-κB/Nrf2 in Acute Liver Injury

**DOI:** 10.3390/toxics12120893

**Published:** 2024-12-09

**Authors:** Minghan Wang, Bin Wu, Kaiyang Tang, Xuexin Wang, Xinyan Liu, Yinan Duan, Jiayu Wang, Xiaoguang Wang, Yinyin Wang, Jun Li, Chenxi Cao, Fangli Ren, Zhijie Chang

**Affiliations:** 1 State Key Laboratory of Membrane Biology, School of Medicine, Tsinghua University, Beijing 100084, China; minghanwang@mail.tsinghua.edu.cn (M.W.); zhijiec@tsinghua.edu.cn (Z.C.); 2 Department of Surgery, The Second Affiliated Hospital of Jiaxing University, No. 397, Huangcheng North Road, Jiaxing 314000, China; gdwkwb@zjxu.edu.cn (B.W.);; 3 Medical School of Chinese PLA, Chinese PLA General Hospital, Beijing 100853, China; 4 Jinfeng Laboratory, No. 313 Jinyue Road, High-Tech Zone, Chongqing 401329, China

**Keywords:** acute liver injury, CREPT, oxidative stress, NF-κB/Nrf2

## Abstract

Background and aims: Cell-cycle-related and expression elevated protein in tumor (CREPT, also named RPRD1B) is highly expressed in tumors and functions to promote tumorigenesis. However, the role of CREPT in the pathophysiology of acute liver injury is limited. Here, we demonstrate that CREPT plays an essential role during acute liver injury. Approach and results: Hepatocyte-specific CREPT knockout (*CREPT^hep−/−^*) and *CREPT^flox/flox^* mice were generated and subjected to the CCl_4_ challenge for the acute (24 h) liver injury. The acute CCl_4_ challenge triggered increased inflammation as well as liver injury, associated with stronger apoptotic and necroptotic cell death in *CREPT^hep−/−^* mice. CREPT knockout down-regulated the expression of different genes involved in cell survival, inflammation and fibrosis under acute CCl_4_ challenge conditions. Antioxidant enzymes such as superoxide dismutase 2 (Sod2) and ferritin heavy chain 1 (Fth1) are dramatically induced at 24 h post-CCl_4_ treatment, but this induction is blocked by transcriptional inactivation of NF-κB/Nrf2, indicating that CREPT might promote hepatocyte survival in acute liver injury by participating in the transactivation of antioxidant genes. Conclusions: These results elucidate the role of CREPT in acute liver injury and provide hints for future research on how CREPT might function in hepatocyte renewal.

## 1. Introduction

Liver injury is widely mimicked in experimental mouse models by the administration of CCl_4_. For acute injury, a high dose of CCl_4_ (0.8–2 μL/g) is applied up to 24 h, and for chronic injury, low doses of CCl_4_ (0.5–0.7 μL/g) are continuously used for extended times (usually after 28 days) in mice [1,2]. CCl_4_ mediates hepatotoxicity through its biotransformation by cytochrome P450 (CYP450) enzymes in hepatocytes, leading to the generation of trichloromethyl radical (CCl3*). This radical can combine with molecular oxygen to form trichloromethylperoxy radical (CCl3OO*), both of which initiate lipid peroxidation and damage the plasma membrane of hepatocytes, ultimately causing necrosis [3]. This leads to the ROS production to destroy the mitochondrial membrane and the electron transport chain [4]. The over-accumulation of ROS further induces hepatocytes to undergo apoptosis/necrosis at 6 h after the acute CCl_4_ challenge [5]. With the time processes, apoptosis/necrosis in centrilobular areas of the liver reaches a peak at 24–48 h after the challenge [6,7]. Simultaneously, ROS activates the JNK (c-Jun N-terminal kinase) and NF-κB (nuclear factor kappa-B) pathways [8] to induce the expression of inflammatory genes [9], and also the anti-apoptotic and anti-oxidant genes including Sod2 (superoxide dismutase 2) and Fth1 (ferritin heavy chain 1) [10]. Both inflammatory factors and antioxidant enzymes activate the proliferation of hepatocytes and stem cells for the recovery of injured livers. After 2–3 days, the acute damages are completely repaired. Under the chronic CCl_4_ challenge condition, the damaged hepatocytes undergo less immediate necrosis/apoptosis but remain damaged to boost inflammation responses [11,12]. Inflammatory factors including TNF-α, IL-1β, and IL-6 are continuously produced to maintain the inflammation environment for activating stellate cells to initiate fibrosis [13,14,15]. In the late stage, fibrogenesis dominates in the liver and ultimately a fibrotic liver is generated [16,17]. Overall, the inflammation factors in particular the abundantly secreted cytokines induce the compensated proliferation of hepatocytes under the acute challenge [18]. Since CCl_4_ exposure represented acute liver injuries, we employed these models to investigate the role of our previously identified factor, CREPT, in liver diseases.

CREPT (cell cycle-related and expression elevated protein in tumor, also known as RPRD1B) is a protein that was initially discovered in cancer cells [19]. CREPT mainly functions as a transcription activator to accelerate the cell cycle and promote tumor growth [20]. Several studies demonstrated that CREPT is highly expressed in a variety of cancers including liver cancer. But the role of CREPT on hepatocytes is unclear, especially under acute challenge. The regulation of CREPT on transcription has been attributed to its role in interacting with RNA polymerase II, p300, β-catenin/TCF4, STAT3 (signal transducer and activator of transcription 3), and other transcriptional regulators [19,21,22]. In this study, we report that CREPT upregulates the antioxidant genes via activation of NF-κB/Nrf2 in acute liver injury. We find that conditional knockout of CREPT in hepatocytes increases the acute liver injury but almost recovers in 48 and 72 h.

## 2. Materials and Methods

### 2.1. Animal Models and Treatments

Mice were kept according to the animal protocol of Laboratory Animal Resources Center, Tsinghua University. All experiments were approved by the Ethics Committee of Tsinghua University (protocol code THU-02-2024-0124A and February 2024 of approval) in accordance with ARRIVE guidelines 2.0 (https://arriveguidelines.org/arrive-guidelines, accessed on 20 January 2024). Hepatocyte-specific CREPT knockout mice (*Alb-Cre*^+/−^;*CREPT^flox/flox^*) were generated by a Cre/loxP system based on the C57BL/6 mouse line. Mice engineered with albumin-promoter-driven Cre, which was specifically expressed in hepatocytes (*Alb-Cre*^+/−^), were used to cross the mouse strain (*CREPT^flox/flox^*) that *CREPT* exon 3 was flanked by loxP sites as previously reported [23,24]. Mice with the genotype of *Alb-Cre*^+/−^;*CREPT^flox/flox^* were used as hepatocyte-specific *CREPT* knockout mice (denoted as *CREPT^hep−^^/^^−^* mice) for the experiment with a control from *CREPT^flox/flox^* mice.

Genotyping of *Alb-Cre* mice was performed using a accessed on PCR protocol with forward primers for *Alb-Cre*: GAA GCA GAA GCT TAG GAA GAT GG, for wild-type: TGC AAA CAT CAC ATG CAC AC, and a common reverse primer TTG GCC CCT TAC CAT AAC TG (5′→3′). PCR was performed under the condition of pre-denaturation at 94 °C for 3 min; 35 cycles of denaturation at 94 °C for 30 s, annealing at 56 °C for 30 s, and extension at 72 °C for 1 min; and final extension at 72 °C for 5 min. The amplified fragment is expected at 351 bp for the wild-type allele and ~390 bp for the *Alb-Cre* transgene allele. A ~150 bp of the fragment was amplified commonly together with the 390 bp fragment in *Alb-Cre* (*CREPT^hep−^^/^^−^*) mice and only 351 bp of the fragment was detected in the wild-type mice (*CREPT^flox/flox^*).

Genotyping of *CREPT^flox/flox^* mice was performed using a PCR protocol with primers: forward TGT ATA GGA TTG TAT TTA AAT TGA TG; reverse GGG AAG CCT GTA TGA CTG GT (5′→3′). The PCR program was set at the condition of pre-denaturation at 94 °C for 3 min; 35 cycles of denaturation at 94 °C for 35 s, annealing at 54 °C for 30 s, extension at 72 °C for 30 s; and final extension at 72 °C for 6 min. The amplified fragment is expected at 284 bp for the wild-type allele and 326 bp for the floxed allele.

### 2.2. Induction of Liver Injury

Male and female C57BL/6 mice with the *CREPT^flox/flox^* or *CREPT^hep−^^/^^−^* genotype of age 4~5 weeks were challenged by intraperitoneal (i.p.) injection with 1 μL/g of CCl_4_ up to 24 h (n = 3), 48 h (n = 4), and 72 h (n = 5~6) for the acute liver injury. Control mice were treated with i.p. injection of the same dose of corn oil. Mice were sacrificed by carbon dioxide over isoflurane to collect blood and livers at 24, 48, and 72 h after the CCl_4_ or corn oil challenge.

### 2.3. Measurement of Serum ALT and AST

Serums were collected at 24, 48, and 72 h after liver injury induction. Blood was allowed to clot at room temperature for 1 h and then centrifuged at 4 °C at 5000 rpm for 10 min. The supernatant was collected as sample serum which was stored at −80 °C for further analyses. The ALT, AST and LDH activities were determined by an automatic biochemical analyzer (Charles River Experimental Animal Technology Limited Corporation, Beijing, China).

### 2.4. Preparation of Histological Slices and Staining

Liver was carefully dissected and immediately immersed in ice-cold PBS. The biggest hepatic lobe was used for histology analyses. After rinsing in PBS, the lobe sample was fixed in 4% PFA (paraformaldehyde) for more than 24 h and embedded in paraffin (Uptbio Biological Technology Limited Corporation, Changsha, China). The tissue was sectioned into 5 μm slices for staining. H&E staining was performed according to the protocol using the H&E stain kit (Solarbio Life Sciences, Beijing, China). All samples were stained under the same condition, and representative images were shown. TUNEL staining was performed according to the protocol using the TUNEL Bright Green Apoptosis Detection Kit (Vazyme, Nanjing, China) and co-stained with DAPI. Negative and positive controls were included. The percentage of TUNEL-positive cells was calculated by the ratio of TUNEL-positive cell number against DAPI-positive cell number. TUNEL-positive and DAPI-positive cells were quantified by Image J.2.1.0.

### 2.5. Protein Isolation and Western Blot

Half of the two hepatic lobes beside the bile duct and gall bladder were used for protein isolation. After sufficient wash in cold PBS, the tissue was placed in 1.5 mL tubes and 150 μL of RIPA buffer (with proteinase inhibitor cocktail) was added. The tissue was sufficiently ground and RIPA was added to make up 500 μL volume in each tube. The lysate was kept at 4 °C on rotation overnight and centrifuged at 4 °C and 12,000 rpm for 10 min. The supernatant was used with 2× loading buffer for Western blot analyses.

Primary antibodies against β-Actin (1:1000, sc-47778, Santa Cruz, CA, USA), 3E10 (1:500, produced by our lab) [23,24] were used with secondary antibodies including goat anti-mouse IgG and goat anti-rabbit IgG (1:10,000, ZSbio, Beijing, China), respectively. The membrane was blocked in 5% skimmed milk in TBST, incubated with indicated antibodies, and exposed to Amersham ECL Prime (Cytiva, Beijing, China). Band density was quantified with ImageJ.

### 2.6. RNA Isolation and PCR Analyses

Half of the two hepatic lobes beside the bile duct and gall bladder were used for RNA isolation using TRIzol reagent (Invitrogen, Carlsbad, CA, USA). RNA was isolated according to a previously reported protocol [25] and the concentration was determined by Nanodrop (Thermo Fisher, Waltham, MA, USA). RNAs were reverse-transcribed by FastKing RT kit (With gDNase) according to the manufacturer’s protocol (Tiangen Biotech, Beijing, China). The genomic DNA was removed by DNase buffered with RNase-free ddH_2_O. The reverse transcription reaction for cDNA synthesis was subject to the condition of 10x King RT Buffer 10 μL, FastKing RT Enzyme Mix 5 μL, FQ-RT Primer Mix 2 μL, with RNase-free ddH_2_O in a volume of up to 50 μL.

Primers for real-time quantitative PCR (qPCR) were designed according to PrimerBank [25]. The primer specificity and self-complementarity were determined by Primer-BLAST (ncbi.nlm.nih.gov, accessed on 20 January 2024). The primers used were Cyp2e1 forward GGA CCT TTC CCA ATT CCT TTC TT, Cyp2e1 reverse TCT TGT GGT TCA GTA GCA CCT; Sod2 forward TGG ACA AAC CTG AGC CCT AAG, Sod2 reverse CCC AAA GTC ACG CTT GAT AGC; Fth1 forward CAA GTG CGC CAG AAC TAC CA, Fth1 reverse GCC ACA TCA TCT CGG TCA AAA (5′→3′). Primers were synthesized by Tsingke Biological Technology Limited Corporation, Beijing, China. qPCR was performed using Talent qPCR PreMix (SYBR Green) kit (Tiangen Biotech, Beijing, China). The qPCR system was set at the condition of 2× Talent qPCR PreMix 10 μL, forward primer (10 μM) 0.6 μL, reverse primer (10 μM) 0.6 μL, cDNA 1.5 μL in a volume of up to 20 μL with RNase-free ddH_2_O. qPCR was performed on Roche Light Cycler 480 (Roche, Mannheim, Germany) with the program of pre-denaturation at 95 °C for 5 min; 40 cycles of denaturation at 95 °C for 20 s, annealing at 60 °C for 20 s, and extension at 72 °C for 30 s. Melting curve analysis was performed for the quantitative presentation of the gene expression by ΔΔCt quantification method. All results were technically repeated 3 times. *GAPDH* was used as an internal control.

### 2.7. RNA-Seq Analysis

Total RNA isolated from hepatic lobes was used for trypsin digestion. RNA purity and quantification were evaluated using the NanoDrop 2000 spectrophotometer (Thermo Scientific, Waltham, MA, USA). RNA integrity was assessed using the Agilent 2100 Bioanalyzer (Agilent Technologies, Santa Clara, CA, USA). Then, the libraries were constructed using VAHTS Universal V6 RNA-seq Library Prep Kit according to the manufacturer’s instructions. The transcriptome sequencing and analysis were conducted by Meiji Biotech Co., Ltd. (Beijing, China). Differentially expressed proteins in different groups were calculated by *p*-value < 0.05, FC (fold change based on control vs. CREPT knockout mice) ≥1.5 or FC ≤1/1.5 and presented as heat maps using R-studio (version 4.0.4). The expression matrix was regressed by linear fitting and the difference in gene expression was calculated by the empirical Bayes method. The total genes with *p*-value < 0.01 were identified as differentially expressed genes. GO and KEGG analyses were performed for the functions of the different genes and the results were presented in volcanic graphs, heat maps and Venn graphs [26,27].

### 2.8. Statistical Analysis

Statistical significance was calculated by a two-tailed parametric *t* test. *p* < 0.05 is determined as significant. Graphs were plotted using GraphPad Prism9.

## 3. Results

### 3.1. CREPT Is Specifically Depleted in Hepatocytes

**Figure 1:**
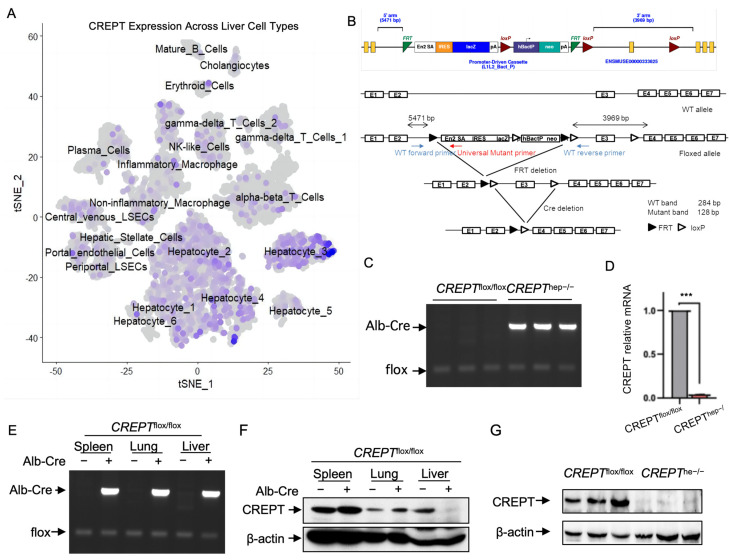
CREPT is specifically knocked out in the hepatocytes of *CREPT^hep^^−/−^* mice. (**A**) CREPT expression in different cell types of the liver, analyzed from a published single-cell RNA-seq data set GSE115469. (**B**) The scheme of hepatocyte-specific CREPT knockout with Alb-Cre/loxP system. (**C**) DNA PCR of *CREPT^flox/flox^* and *CREPT^hep−/−^* male mice. (**D**) qPCR confirmed CREPT knockout in *CREPT^hep−/−^* male mice. Density ratio was the band density of CREPT divided by that of GAPDH. (**E**) DNA PCR of mouse spleen, lung, and liver in *CREPT^flox/flox^* and *CREPT^hep−/−^* male mice. (**F**) Western blot of mouse spleen, lung, and liver in *CREPT^flox/flox^* and *CREPT^hep−/−^* male mice. (**G**) Western blot confirmed the hepatocyte-specific ablation of the CREPT protein in homozygous *CREPT^hep−/−^* male mice. ***, *p* < 0.001 by two-tailed *t* test. Error bar: mean ± SEM (standard error of measurement).

Previous reports have demonstrated that CREPT is highly expressed in hepatocytes of liver cancer. Thus, we sought to explore the detailed role of CREPT in the physiological and pathological processes of the liver. Firstly, we analyzed the expression of CREPT across different liver cell types in a published single-cell RNA-seq dataset (GSE115469) [28]. Interestingly, we observed that the CREPT mRNA level was predominantly high in hepatocyte clusters (Figure 1A). Therefore, we generated hepatocyte-specific CREPT knockout mice for further research. A transgenic mouse with loxP sites flanking *CREPT* exon 3 was generated and then crossed with an albumin-Cre mouse, resulting in progeny with CREPT depleted in hepatocytes, hereafter referred to as *CREPT^hep−^^/^^−^* (*Alb-Cre*^+/−^; *CREPT^flox/flox^*) (Figure 1B). After genotyping, we measured the expression level of CREPT. To further test the efficiency of CREPT knockout in the liver, we performed PCR and RT-qPCR on *CREPT^flox/flox^* and *CREPT^hep−^^/^^−^* mice. The results show that CREPT expression is dramatically decreased in the livers of *CREPT^hep−^^/^^−^* mice (Figure 1C,D). To confirm the specificity of CREPT knockout in the liver, we detected *Alb-Cre* and *flox* alleles and CREPT expression in the spleen, lung, and liver. As evidenced by the results, CREPT is specifically depleted in the liver (Figure 1E,F). Consistently, we observed that the protein level of CREPT, by a Western blot analysis, was dramatically reduced in the liver of *CREPT^hep−^^/^^−^* mice (Figure 1G).

### 3.2. Hepatocyte-Specific CREPT Knockout (CREPT^hep−/−^) Mice Demonstrated No Defect in the Liver Development

**Figure 2:**
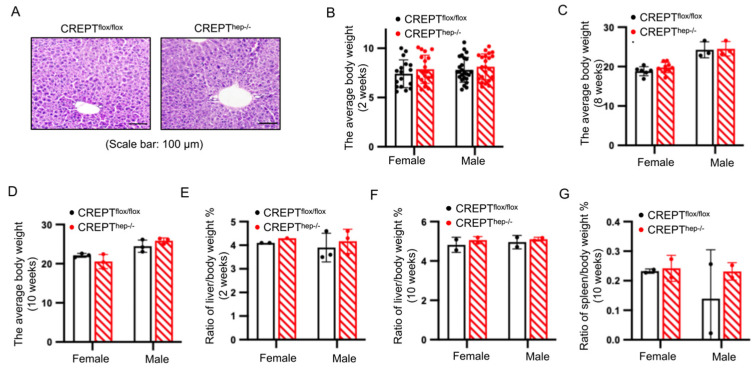
Hepatocyte-specific CREPT knockout (*CREPT^hep^^−/−^*) mice demonstrated no defect in liver development. (**A**) H&E staining of liver from *CREPT^flox/flox^* and *CREPT^hep−/−^* male mice 14 days after birth (n = 4). (**B**) Average body weight at 2 weeks after birth in female (*CREPT^flox/flox^* = 17, *CREPT^hep−/−^* = 19) and male mice (*CREPT^flox/flox^* = 25, *CREPT^hep−/−^* = 22). (**C**) Average body weight at 8 weeks after birth in female (*CREPT^flox/flox^* = 6, *CREPT^hep−/−^* = 6) and male mice (*CREPT^flox/flox^* = 3, *CREPT^hep−/−^* = 3). (**D**) Average body weight at 10 weeks after birth in *CREPT^flox/flox^* and *CREPT^hep−/−^* mice (female and male, n = 3). (**E**) The ratio of liver weight to body weight *CREPT^flox/flox^* and *CREPT^hep−/−^* mice at 2 weeks after birth (female and male, n = 3). (**F**) The ratio of liver weight to body weight of in *CREPT^flox/flox^* and *CREPT^hep−/−^* mice at 10 weeks after birth (female and male, n = 2). (**G**) The ratio of spleen weight to body weight of in *CREPT^flox/flox^* and *CREPT^hep−/−^* mice at 10 weeks after birth (female and male, n = 2). Error bar: mean ± SEM (standard error of measurement).

To examine the phenotype of *CREPT^hep−^^/^^−^* mice, we maintained the mouse line under the general raising condition and observed that the mice developed normally without any defect in comparison with *CREPT^flox/flox^* mice. We then characterized the mice at the adult age. A histological analysis using H&E (hematoxylin and eosin) staining showed that livers of both *CREPT^hep−^^/^^−^* and *CREPT^flox/flox^* mice were of normal structures, including the complete hepatic lobule and regularly arranged hepatic cords (Figure 2A). To further assess the development of the mice by measuring the body and organ weights, we included both male and female mice. The result showed no statistically significant difference in the body weights between the female and male *CREPT^hep−^^/^^−^* and *CREPT^flox/flox^* mice at 2 weeks (Figure 2B), 8 weeks (Figure 2C) and 10 weeks (Figure 2D) after the birth. Furthermore, we observed no statistical difference in the ratio of liver weight to body weight between *CREPT^hep−^^/^^−^* and *CREPT^flox/flox^* mice at 2 weeks (Figure 2E) and 10 weeks after birth (Figure 2F). Simultaneously, the ratio of spleen weight to body weight did not change between *CREPT^hep−^^/^^−^* and *CREPT^flox/flox^* mice at 10 weeks after birth (Figure 2G). It is also mentionable that the conditional knockout mice reproduce normally. All these results suggest that conditional knockout of CREPT influences neither liver nor whole body development from birth to adulthood.

### 3.3. CREPT Knockout Exacerbates Liver Injury Under the Acute Challenge

**Figure 3:**
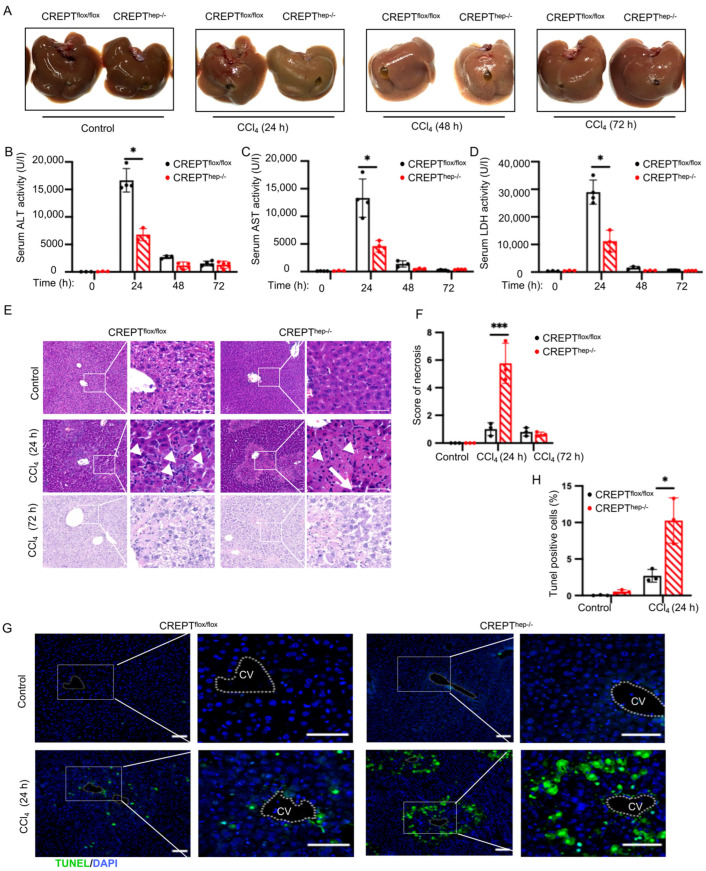
CREPT knockout exacerbates liver injury under the acute challenge. (**A**) *CREPT^hep−/−^* and *CREPT^flox/flox^* mice were exposed to a single injection of CCl_4_ 24 h (n = 6–8). (**B**–**D**) Serum ALT, AST, LDH activity in *CREPT^hep−/−^* and *CREPT^flox/flox^* mice at 0 h, 24 h, 48 h, 72 h CCl_4_ treatment (n = 4). (**E**) H&E staining of *CREPT^hep−/−^* and *CREPT^flox/flox^* livers at 0 h, 24 h, 72 h CCl_4_ treatment. At 24 h after CCl_4_ treatment, hepatocytes of *CREPT^flox/flox^* mice show signs of apoptosis (arrows), while hepatocytes of *CREPT^hep−/−^* livers show signs of inflammatory infiltration, apoptosis and necrosis (arrows). (**F**) Statistical of necrosis in *CREPT^hep−/−^* and *CREPT^flox/flox^* mice at 0 h, 24 h, 72 h CCl_4_ treatment. (**G**) TUNEL staining of *CREPT^hep−/−^* and *CREPT^flox/flox^* livers at 24 h CCl_4_ treatment. (**H**) The percentage of TUNEL^+^ cells was represented by the ratio of TUNEL^+^ cells to DAPI^+^ cells. Scale bar: 50 μm. DAPI: 4′,6-diamidino-2-phenylindole. CV: central vein. *, *p* < 0.05, *** *p* < 0.001, by two-tailed *t* test. Error bar: mean ± SEM (standard error of measurement).

As the CREPT conditional knockout mice developed normally under the stable raising condition, we questioned whether the mice had any response under pathological conditions. To this end, we decided to challenge the male mice to induce acute liver injuries. We first investigated the effect of CREPT on acute liver injury by using intraperitoneal injection with a high dose (1 μL/g) of CCl_4_ for 24 h. The result showed that livers from *CREPT^hep−^^/^^−^* and *CREPT^flox/flox^* mice had no difference as observed by the sizes and appearances before and after the acute CCl_4_ challenge for 24 h (Figure 3A, first and second sets of panels). However, we observed that both livers from *CREPT^hep−^^/^^−^* and *CREPT^flox/flox^* mice appeared pale at 48 h but recovered into normal dark-reddish color at 72 h after the acute CCl_4_ challenge (Figure 3A, right two sets of panels). Interestingly, a biochemistry analysis showed that serum ALT (alanine aminotransferase) (Figure 3B), AST (aspartate aminotransferase) (Figure 3C), and LDH (lactate dehydrogenase) (Figure 3D) were greatly increased in *CREPT^flox/flox^* mice at 24 h and declined to the basal level at 48 and 72 h after the acute CCl_4_ challenge. Furthermore, the increased levels of these biochemistry indices were attenuated in *CREPT^hep−^^/^^−^* mice (Figure 3B–D, red columns). We then addressed whether knockout of CREPT in hepatocytes causes any defect in the histological structures of the liver under the acute CCl_4_ challenge, as we observed no alteration in the liver structures between *CREPT^hep−^^/^^−^* and *CREPT^flox/flox^* mice in the normal condition (see Figure 3A, top panels). An H&E staining analysis showed that the histological liver structure of *CREPT^hep−^^/^^−^* mice was quite different from that of *CREPT^flox/flox^* mice at 24 h after the acute CCl_4_ challenge (Figure 3E, bottom panels). In particular, we observed that the centrilobular area was more severally damaged in *CREPT^hep−^^/^^−^* mice than in *CREPT^flox/flox^* mice after the acute CCl_4_ challenge (Figure 3E, bottom panel, boxed regions). Enlarged images demonstrated that the damaged structure reflected the apoptosis and/or massive necrosis of hepatocytes as the cells were stained with strong eosinophilic cytoplasm (Figure 3E, arrows). A quantitative examination showed that the apoptosis/massive necrosis areas were greatly increased in the liver of *CREPT^hep−^^/^^−^* mice in comparison with the liver of *CREPT^flox/flox^* mice under the acute challenge (Figure 3F). Clearly, many more inflammatory cells were infiltrated in the death regions in the liver of *CREPT^hep−^^/^^−^* mice than in the liver of *CREPT^flox/flo^*^x^ mice after the acute CCl_4_ challenge (Figure 3E, see the smaller cells marked with arrows). These results suggest that knockout of CREPT in hepatocytes induces more inflammation and apoptosis/necrosis under the acute challenge. Interestingly, we observed that the histological alterations at 24 h after the acute CCl_4_ challenge were almost completely diminished at 72 h (Figure 3E, bottom panels, Figure 3F, right panels). This recovered histological structure is consistent with the decreased levels of ALT, AST and LDH in the serum of the mice after the acute CCl_4_ challenge. Since the occurrence of apoptosis/necrosis reflects an ability of liver compensation reactions upon toxicity, we reason that CREPT knockout-induced hepatic deaths are in favor of the detoxification and recovery against CCl_4_. To confirm the apoptosis, we performed a TUNEL staining experiment. The result showed that the apoptosis occurred in the centrilobular area around the central vein in *CREPT^flox/flox^* mice after the acute CCl_4_ challenge (Figure 3G, left bottom panel), similar to the hepatic death regions as observed by the H&E staining. Consistently, the apoptotic cells were much more in the liver of *CREPT^hep−^^/^^−^* mice than that of *CREPT^flox/flox^* mice challenged by CCl_4_ (Figure 3G, compare right and left panels). A statistical analysis showed that the TUNEL-positive cell numbers were greatly increased in the acute CCl_4_-challenged *CREPT^hep−^^/^^−^* mice (Figure 3H). These results indicate that conditionally knocking out CREPT increases apoptosis/necrosis of hepatocytes in response to the acute CCl_4_ challenge, but has no effect on the liver under the normal raising condition. Taken together, all the results suggest that CREPT knockout exacerbates hepatic death but decreases ALT and AST levels in the serum in the acute CCl_4_ challenge.

### 3.4. CREPT Upregulates the Expression of Inflammation Genes in Response to the Acute CCl_4_ Challenges

**Figure 4:**
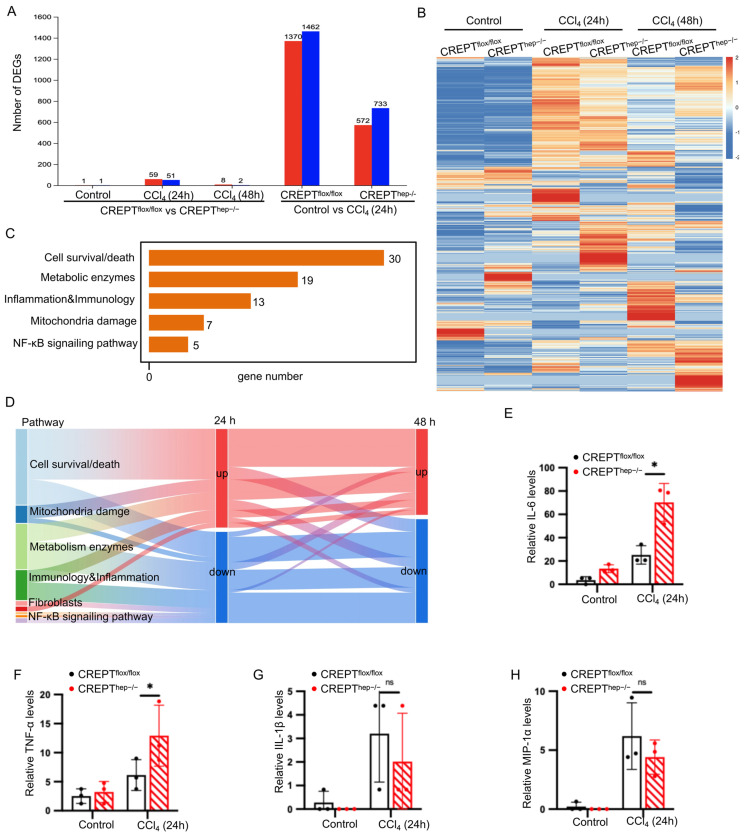
CREPT upregulates the expression of inflammation genes in response to the acute CCl_4_ challenges. CREPT upregulates the antioxidant genes via activation of NF-κB/Nrf2 signal transcription in acute liver injury. (**A**) RNA-seq experiment with the mice at 0 h, 24 h and 48 h after the acute CCl_4_ challenge. Based on the quantitative results of expression, the differentially expressed genes between *CREPT^flox/flox^* and *CREPT^hep−/−^* livers were analyzed to obtain the differentially expressed genes. The screening threshold was |log2FC| ≥ 1 & *p*-value < 0.05. (**B**) Heat map of the differentially expressed genes between *CREPT^flox/flox^* and *CREPT^hep−/−^* livers at 0 h, 24 h and 48 h after the acute CCl_4_ challenge. (**C**) KEGG enrichment analysis of differentially expressed genes at 24 h after CCl_4_ induction. (**D**) The Sankey plots of differentially expressed genes between *CREPT^flox/flox^* and *CREPT^hep−/−^* livers at 0 h, 24 h and 28 h. (**E**–**H**) Elisa analysis of IL-6, TNF-α, IL-1β and MIP-1α between *CREPT^flox/flox^* and *CREPT^hep−/−^* livers after acute CCl_4_ challenge. *, *p* < 0.05, by two-tailed *t* test. ns, not significant by two-tailed *t* test. Error bar: mean ± SEM (standard error of measurement).

Our aforementioned results indicated that CREPT knockout in the hepatocytes exacerbated the hepatic death in response to the acute CCl_4_ challenge but ameliorated in 48 and 72 h. To unravel why CREPT plays different roles between acute and chronic challenges, we determined to examine the gene expression alteration of the liver. We first performed an RNA-seq experiment with the mice at 24 h, 48 h and 72 h after the acute CCl_4_ challenge. The result showed that 51 genes were downregulated and 59 genes were upregulated at 24 h after the acute challenge (Figure 4A). Interestingly, we observed only 10 markedly changed genes in CREPT knockout mice at 48 h after the acute CCl_4_ challenge (Figure 4A), indicating that the gene expression alterations by the acute CCl_4_ challenge were decreased with the time of liver recovery after its damage. On the other hand, we observed that 1370 genes were upregulated and 1462 genes were downregulated in the wild-type liver but 572 genes were upregulated and 733 genes downregulated in the CREPT knockout liver at 24 h after the acute CCl_4_ challenge (Figure 4A, right panels). These different gene expression patterns were illustrated in a heat map analysis (Figure 4B). We focused on the genes with alterations (59 and 51) between *CREPT^flox/flox^* and *CREPT^hep−^^/^^−^* mice at 24 h after the acute CCl_4_ challenge. A KEGG and Hohsandhorn analysis indicated that these genes were involved in cell survival/death, metabolism, inflammation responses and NF-κB signaling pathway (Figure 4C,D). These alterations echo the phenotype that the acute challenge induces more cell death under CREPT knockout. Taken together, all the RNA-seq analyses suggest that knockout of CREPT in hepatocytes influences the expression of genes involved in the different biological processes including the maintenance of cell survival and the inflammation responses. We then performed Elisa assays to examine the expression of inflammatory factors. The result showed that the acute CCl_4_ challenge increased the levels of IL-6, TNF-α, IL-1β, and MIP-1α in the wild-type mice (*CREPT^flox/flox^*) (Figure 4E–H, black columns). Interestingly, we observed that CREPT knockout further increased IL-6 and TNF-α significantly (Figure 4E,F) but not IL-1β and MIP-1α (Figure 4G,H). As IL-6 and TNF-α have been acknowledged for the induction of cell death, we attribute the CREPT knockout enhanced production of these two factors to the increased cell death phenotype in the acute CCl_4_ challenge.

**Figure 5:**
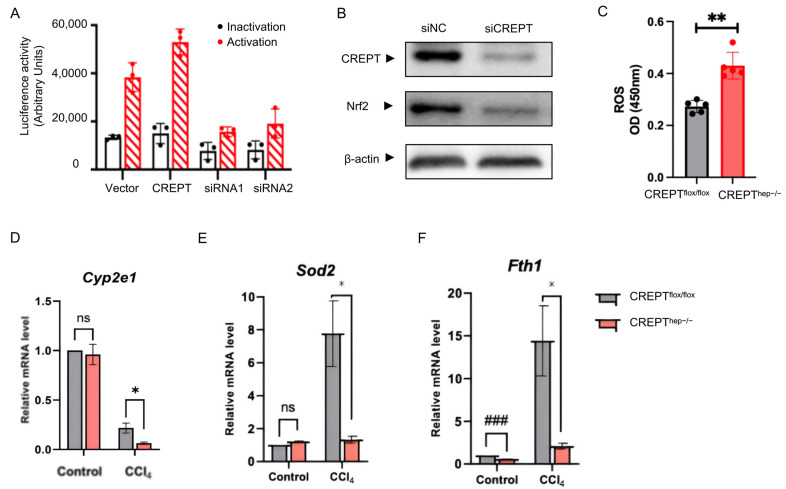
CREPT upregulates the antioxidant genes via activation of NF-κB/Nrf2 Transcription in acute liver injury. (**A**) Overexpression CREPT and knocking down CREPT by siRNAs promote and abolish the luciferase activities of the NF-κB driven reporter. (**B**) Nrf2 was decreased after knocking down CREPT by siRNAs. (**C**) OD450 of ROS in livers from *CREPT^flox/flox^* and *CREPT^hep−/−^* male mice after acute CCl_4_ challenge. (**D**–**F**) RT-PCR of Cyp2e1, Sod2 and Fth1 in livers from *CREPT^flox/flox^* and *CREPT^hep−/−^* male mice under normal condition and acute CCl_4_ challenge. *, *p* < 0.05, **, *p* < 0.01 by two-tailed *t* test as significant. Compared within the control group, ### *p* < 0.001, ns, not significant by two-tailed *t* test. N = 3. Error bar: mean ± SEM (standard error of measurement).

KEGG and Hohsandhorn analysis indicated that the differentially expressed genes in CREPT knockout mice were mainly enriched in NF-κB which can be activate by inflammatory factors. To this end, we performed a luciferase reporter experiment using the NF-κB response element. The result showed that over-expression of CREPT promoted the luciferase activity upon NF-κB activation (Figure 5A, left panels). Reciprocally, we observed that knocking down CREPT by siRNAs almost abolished the luciferase activities of the NF-κB driven reporter (Figure 5A, right panels). Given that Nrf2 is a well-known regulator of antioxidant genes, we tested the endogenous level of Nrf2 in a cell line. We observed that the expression of Nrf2 was significantly decreased when CREPT was knocked down by siRNA (Figure 5B). These results corroborate our hypothesis that CREPT is involved in the up-regulation of NF-κB target genes by interacting with phosphorylated RelA and promoting RelA/p50 complex formation. All of these results illustrate a scenario in which CREPT promotes the inflammation response by regulating the transcription of gene expression. Simultaneously, we performed RT-PCR analyses to examine the expression of genes related to hepatocyte metabolism. The result showed that Cyp2e1, an oxidoreductase preferentially expressed in hepatocytes in the centrilobular area [29], has no obvious change by the acute CCl_4_ challenge in both wild-type and CREPT hepatocytes knockout mice (Figure 5C). As CCl_4_ is metabolically transferred by CYP450 enzymes into radicals CCl3* and CCl3OO*, which subsequently leads to massive ROS production to induce cell death [4], the liver always expresses genes to compensate for ROS. In this context, we determined to examine another class of enzymes related to mitochondria. We focused on sod2 and fth1, two enzymes that counteract ROS during CCl_4_-mediated oxidative stress [30,31]. The result showed that both Sod2 and Fth1 were upregulated about 8-fold and 15-fold upon the acute CCl_4_ challenge in the wild-type livers (Figure 5D,E, grey columns) but remained at the basal levels in *CREPT^hep−^^/^^−^* mice (Figure 5D,E, red columns). Taken together, CREPT knockout might abrogate Sod2 and Fth1 upregulation by decreasing the transcriptional activity of NF-κB/Nrf2. When CREPT is depleted, antioxidant enzymes like Sod2 and Fth1 cannot be sufficiently induced by activated NF-κB/Nrf2, resulting in accumulated ROS and exacerbated hepatocellular injury. We reason that the severely damaged liver phenotype is due to the loss of hepatocyte survival and compensation gene expression upon the acute CCl_4_ challenge.

**Figure 6:**
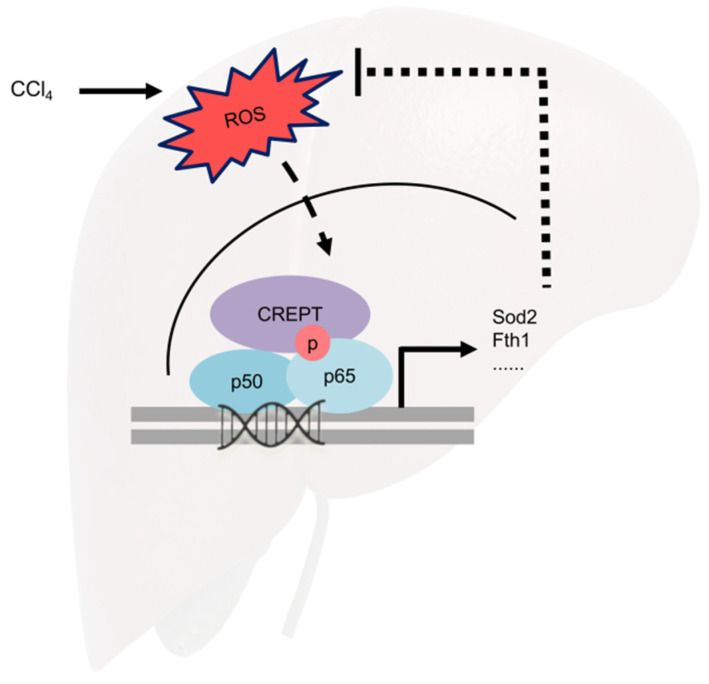
Schematic of CREPT-driven antioxidant genes upregulation via NF-κB activation in acute liver injury.

Taken together, CREPT knockout might abrogate Sod2 and Fth1 upregulation by decreasing the transcriptional activity of NF-κB/Nrf2. When CREPT is depleted, antioxidant enzymes like Sod2 and Fth1 cannot be sufficiently induced by activated NF-κB/Nrf2, resulting in accumulated ROS and exacerbated hepatocellular injury (Figure 6).

## 4. Discussion

CREPT, a previously identified pro-tumorigenic protein, has been widely reported by both our group and others [20]. In this study, we observed that knockout of CREPT in hepatocytes had a minimal impact on mice under normal raising conditions but elicited distinct responses during acute CCl_4_ challenges. Intriguingly, we observed that knockout of CREPT resulted in severe centrilobular necrosis within 24 h of the acute CCl_4_ challenge. The liver endures severe damage under CCl_4_ exposure, causing rapid and severe damage to hepatocytes leading to necrosis, which subsequently leads to necrosis and apoptosis. Under these conditions, CREPT knockout in hepatocytes exacerbates the damaged phenotype at 24 h but has no influence on liver recovery at 72 h following the acute challenge. Recovery was driven by compensated proliferation and differentiation of stem cells. In light of these findings, we proposed that CREPT may promote hepatocytes to proliferate following damage, and its knockout may induce the rapid onset of apoptosis. Our hypothesis is supported by the results of other studies in which increased apoptosis occurred when CREPT was deleted in cancer cells [32]. In one instance, CREPT participates in the production of the inflammatory factors induced by the damaging effect of CCl_4_ in hepatocytes. This inflammation boosts a series of responses not only in hepatocytes but also in other cells such as macrophages/Kupffer cells and astrocytes/fibroblasts [33]. Therefore, knockout of CREPT reduces the expression of these factors and decreases the fibrosis during the chronic challenge. Since the inflammation occurs following damage, it is conceivable that knockout of CREPT increases the level of inflammation accompanied by increased damage during the acute challenge.

We consider that the knockout of CREPT in hepatocytes has a favorable effect on the liver upon acute challenges. During acute challenge, due to the high dose of CCl_4_ applied, increased necrosis/apoptosis exerts a protective effect on the liver in order to increase its chances of regeneration. Therefore, the elevated necrosis/apoptosis induced by CREPT knockout should be considered a protective mechanism, as demonstrated by the increase in LDH, ALT, and AST levels. It is conceivable that, without necrosis/apoptosis, the liver would remain vulnerable to ongoing damage with the eventual development of fibrosis. Conversely, knockout of CREPT in hepatocytes reduces the expression of inflammatory factors. Our model is also supported by the alteration of genes in the livers of wild-type and CREPT conditional knockout mice under acute challenges. We identified different sets of genes representing the process of cell survival, inflammation and fibrosis. Evidently, during acute challenge, the genes that undergo the most significant changes are those involved in cell survival; however, another set of inflammation-related genes also remains altered. The major enzyme that converts CCl_4_ into hepatotoxic radicals is Cyp2e1 [29], as Cyp2e1 terminates CCl_4_-induced liver injury in mice [30]. In this study, we observed the CREPT knockout resulted in dramatically decreased Cyp2e1 expression under the acute CCl_4_ challenge (see Figure 5D). We consider that the decreased Cyp2e1 expression is due to the damage of hepatocytes induced during acute challenges, which is exacerbated by CREPT knockout. Our results are consistence with the observation of decreased Cyp2e1 mRNA levels in the liver following CCl_4_ treatment at the same dosage used in our study [31]. However, Zhang et al. observed upregulated Cyp2e1 mRNA with a lower dosage of CCl_4_ treatment [30], contrary to our observations and those of Ghafoory et al. In particular, we confirmed the upregulation of Sod2 (superoxide dismutase 2, also known as manganese superoxide dismutase, MnSOD) and Fth1 (Ferritin) in the wild-type liver but not in the CREPT knockout hepatocytes in response to CCl_4_-induced acute liver injury. The levels of these two genes reflect the survival status of hepatocytes to counteract the damage because oxidoreductases are responsible for the maintenance of cellular redox homeostasis. Sod2 is a major ROS metabolizer induced by cellular oxidative stress by catalyzing the dismutation of O_2_^-^* into H_2_O_2_. This reaction represents the first line of cell defense against oxidative stress [34]. Following CCl_4_-induced liver injury, the Sod2 activity is rapidly diminished by oxidation which in turn exacerbates ROS accumulation [14,15]. However, during oxidative stress, Sod2 transcription is stimulated by inflammatory factors such as NF-κB/Nrf2 to eliminate the accumulated ROS and promote cell survival [35]. Fth1 is another protein that contributes to ROS metabolism by binding iron to interrupt the Fenton reaction with H_2_O_2_, which produces highly reactive OH* radicals. Through this mechanism, Fth1 synergizes with Sod2 to eliminate cellular ROS [36]. Similarly, Fth1 is also typically induced by activated NF-κB/Nrf2 in response to oxidative stress [11]. The failure of both Sod2 and Fth1 expression under CREPT knockout implies severe damage and poor survival of hepatocytes. As CREPT knockout increased apoptosis/necrosis of hepatocytes, we reason that the expression of these two genes is directly regulated by CREPT. In this context, the hepatocytes lost the ability to counteract the production of ROS and therefore remained in a status of producing inflammatory factors such as IL-6 and TNF-α under the acute challenge.

*CREPT^hep−^^/^^−^* mice showed an increase in IL-6 and TNF-α but a decrease in IL-1β and MIP-1α under the acute challenge. We reason that IL-6 and TNF-α were directly induced by the severe damage that was exacerbated by CREPT knockout in the acute challenge. This finding is supported by the observation that TNF-α and IL-6 were induced during the impaired hepatocyte regeneration to activate precursor cell proliferation [37,38,39,40]. Previous reports indicated that the expression of inflammatory factors was induced by NF-κB, together with DAMP and priming signals for the inflammation [35]. Indeed, in a luciferase reporter assay using the NF-κB response element, we observed that knockout of CREPT decreased whereas overexpression of CREPT increased transcriptional activity upon NF-κB activation. All of our results provide a scenario in which CREPT promotes the inflammation response by participating in NF-κB activation. The failure of both Sod2 and Fth1 expression under CREPT knockout implies severe damage and poor survival of hepatocytes. In addition, we observed that the levels of Nrf2, another regulator in the Sod2 expression, were decreased when CREPT was knocked down. As CREPT knockout induces increased apoptosis/necrosis of hepatocytes, we reason that the expression of these two genes is directly regulated by CREPT. In this context, the hepatocytes lost their ability to counteract the production of ROS and therefore remained in a status of producing inflammatory factors such as IL-6 and TNF-α under the acute challenge.

In summary, the results of our study highlight the essential role of CREPT in the hepatocellular response to CCl_4_-mediated acute liver injury. Under these conditions, CREPT knockout mice suffer exacerbated liver injury and hepatocyte necrosis, which is likely attributed to decreased transcription of antioxidant enzymes in the absence of CREPT via transcriptional activation of NF-κB/Nrf2. These results provide the foundation for future research into how CREPT functions in hepatocyte renewal.

## Data Availability

RNA-seq data for mice are available in the paper. The expression of CREPT across different liver cell types used in this study was obtained from a published single-cell RNA-seq dataset (GSE115469) [28].
